# Binder-Free Charantia-Like Metal-Oxide Core/Shell Nanotube Arrays for High-Performance Lithium-Ion Anodes

**DOI:** 10.3389/fchem.2020.00159

**Published:** 2020-03-06

**Authors:** Pingping Xu, Ziying Zhang, Huizhen Zhang, Ao Shen, Yangqiang Zhao, Yangyang Zhou, Ying Weng

**Affiliations:** ^1^School of Materials Engineering, Shanghai University of Engineering Science, Shanghai, China; ^2^School of Management, University of Shanghai for Science and Technology, Shanghai, China

**Keywords:** carbon fabric, TiO_2_ nanotube arrays, α-Fe_2_O_3_ hollow nanospheres, lithium-ion batteries, anodes

## Abstract

The performance of anodes of lithium-ion batteries relies largely on the architecture and composition of the hybrid active materials. We present a two-step, seed-free, solution-based method for the direct growth of hierarchical charantia-like TiO_2_/Fe_2_O_3_ core/shell nanotube arrays on carbon cloth substrates. An ultrahigh loading of the nanomaterial on carbon fibers was achieved with this method without the use of a binder. This three-dimensional porous hollow architecture and its direct contact with the CC current collector ensure an efficient electronic pathway. The hollow TiO_2_ framework effectively protects the hierarchical charantia-like TiO_2_/Fe_2_O_3_ hollow core/shell arrays from collapsing because of its negligible volume change during cycling. Meanwhile, the self-assembled α-Fe_2_O_3_ hollow nanospheres guarantee a large capacity and contact area with the electrolyte. This flexible anode with a 3D porous charantia-like hollow architecture exhibits high cycle performance, reversible capacity, and rate capability. These nanotube arrays maintain a high reversible capacity of 875 mAh g^−1^ after 200 cycles at a current density of 200 mA g^−1^. This simple, cost-effective, and scalable electrode fabrication strategy can be implemented in the fabrication of high-performance wearable energy storage devices.

## Introduction

Lithium-ion batteries (LIBs) are popular energy storage devices because of their high energy density, high open-circuit voltage, lack of memory effects, and shape controllability (Joshi et al., 2016; Zhao et al., 2016; Shen et al., 2018; Deng et al., 2019). The design of electrode materials with high energy density and cycling stability is important for enhancing the performance of LIBs. Graphite—the most widely used commercial anode material—cannot fulfill the increasing demands with regard to the energy density and stability of an electrode owing to its low theoretical capacity (372 mAh g^−1^), poor rate performance, and intrinsic stability. Therefore, much attention has been focused on exploring anode materials with higher capacity for next-generation LIBs (Liu et al., 2019; Partheeban and Sasidharan, 2019; Shen et al., 2019).

TiO_2_ is a promising anode candidate owing to its relatively good conductivity, low cost, higher operation voltage, and low volume expansion. However, its practical application is impeded by its low theoretical capacity. Owing to its unique physical and chemical properties as a nanomaterial, TiO_2_ is engineered into various nanostructures with different morphologies to improve the specific capacity (Moretti et al., 2013; Ying et al., 2017; Smith et al., 2019). Despite immense progress in this area, the effect of scaling down on the performance of TiO_2_ has been limited due to its theoretical capacity limitation. Recent studies have indicated that TiO_2_-based nanocomposites obtained by combining them with high-capacity metal oxides could facilitate high performance at a smaller scale (Zhu et al., 2014; Wang et al., 2015; Lee et al., 2017; Yang et al., 2018; Zhang et al., 2019). Hematite (α-Fe_2_O_3_) is a promising mineral for preparing a hybrid with TiO_2_ because of its relatively higher theoretical capacity (~1,000 mA h g^−1^), non-toxicity, high corrosion resistance, and abundant resource (Gao et al., 2014; Li et al., 2016; Jiang et al., 2017; Zhao et al., 2019). When Fe_2_O_3_ is anchored onto the surface of a TiO_2_ nano-backbone, its high theoretical capacity compensates for the deficient capacity of the TiO_2_ nano-backbone, while the good conductivity and low volume expansion of TiO_2_ ensures excellent cyclic stability of the hybrid material (Luo et al., 2012; Yang et al., 2017). One-dimensional (1D) nanotube arrays and three-dimensional (3D) hollow spheres can promote rate capability by offering an efficient electronic pathway (Han et al., 2011; Wang et al., 2012; He et al., 2018). Therefore, we fabricated 3D nanotube arrays by coating Fe_2_O_3_ hollow nanospheres on TiO_2_ nanotube arrays to realize high-performance rechargeable batteries. However, the direct growth of a 3D porous hollow composite architecture on a flexible substrate without a binder remains a major challenge.

In this study, 3D hierarchical charantia-like TiO_2_/Fe_2_O_3_ nanotube arrays were grown directly on a carbon cloth (CC) substrate by a facile hydrothermal method. The CC/TiO_2_ nanotube arrays decreased the fracture risk caused by the volume fluctuation during lithium-ion insertion and extraction, while the α-Fe_2_O_3_ nanosphere shell assembled on the surface of TiO_2_ nanotube increased the surface area and number of active sites. Direct contact of the active material with the CC substrate and the hollow structure facilitated an efficient electronic pathway. Their unique hollow core-shell structure endowed the final electrodes with high energy, reversible capacity, and cycle performance.

## Experimental Method

### Synthesis of TiO_2_ Nanotube Arrays on CC

Before the experiment, the CC substrates were immersed in nitric acid at 30°C for 3 h, and then ultrasonically cleaned with distilled water and ethanol for 30 min. TiO_2_ nanotube arrays were synthesized by a facile hydrothermal method. Analytically pure reagents were used without further purification. Typically, 1 mL of tetrabutyl titanate, 12 mL of glycerinum, and 45 mL of ethanol were magnetically stirred at room temperature to form a homogeneous precursor solution. The precursor was then poured into a 100 mL Teflon-lined autoclave with a piece of clean CC (7 ×5.0 cm) and maintained at 175°C for 20 h. After the reaction, the white byproduct on the CC was washed with ethanol and distilled water, and CC/TiO_2_ nanotube arrays were obtained after heating the products at 350°C for 1 h.

### Growth of Fe_2_O_3_ Nanospheres on CC/TiO_2_ Nanotube Arrays

The as-prepared CC/TiO_2_ nanotube arrays were used as scaffolds for growing α-Fe_2_O_3_ hollow nanospheres via a simple hydrothermal method. FeCl_3_·6H_2_O (1.688 g) and ammonia (0.1 mL) were dissolved in 40 mL of deionized water in a 100 mL Pyrex beaker and stirred to form a clear tan solution. The final mixture was transferred to an 80 mL Teflon-lined autoclave and heated at 95°C for 4 h. The products were washed several times with ethanol and water. The dried products were further annealed in a N_2_ atmosphere at 450°C for 2 h.

### Characterization of the Structure and Morphology

Phase purity of the synthesized products was examined by X-ray powder diffraction (XRD, Panalytical X'Pert, Netherlands) carried out with Cu-*K*α radiation (λ = 1.5418 Å). The morphology of the products was characterized by field-emission scanning electron microscopy (SEM, Model: JEOL JSM-7000F, Japan) and high-resolution transmission electron microscopy (HRTEM, Model: FEI Titan X 60-300, USA). FA1004 electronic balance was used to measure the weight difference between the prepared electrode and CC substrate, and the mass loading of the active material was obtained. X-ray photoelectron spectroscopy (XPS) was performed on a Perkin-Elmer model PHI 5600 XPS system, with a monochromated aluminum anode as the X-ray source. The specific surface area of the obtained products was calculated by the Brunauer–Emmett–Teller (BET) method using the nitrogen adsorption–desorption isotherm acquired from a Micrometics Tristar 3000 system.

### Electrochemical Measurements

Electrochemical tests were performed in CR 2032 coin half-cells assembled in an argon-filled glovebox using the as-prepared hierarchical nanotube arrays as the anode and circular lithium-metal foil as the cathode. A solution of 1 M LiPF_6_ in ethylene carbonate and diethyl carbonate at a volume ratio of 1:1 was used as the electrolyte. The cells were galvanostatically charged and discharged using a multichannel battery tester (Neware-CT3008). With lithium-metal foil as the counter and reference electrodes, Cyclic voltammetry (CV) of the coin half-cells was performed on a PARSTAT 4000 electrochemical workstation at a scan rate of 0.2 mV s^−1^ in the range of 3.0–0.01 V vs. Li/Li^+^. Electrochemical impedance spectroscopy (EIS) was performed in a wide frequency range of 100 kHz to 0.01 Hz at an AC perturbation voltage of 5 mV.

## Results and Discussion

### Synthesis and Characterization

**Scheme 1:**
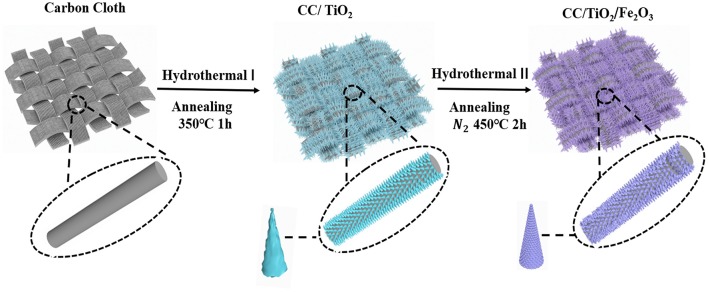
Schematic illustration of the synthesized procedures of CC/TiO_2_/Fe_2_O_3_ hollow nano-arrays.

Scheme 1 shows the synthesis of the 3D hierarchical charantia-like CC/TiO_2_/Fe_2_O_3_ nanotube arrays. First, the TiO_2_ nanotube arrays were fabricated on a CC substrate by a facile seed-free hydrothermal method. After annealing the obtained product in air at 350°C for 1 h, the amorphous TiO_2_ cores crystallized into one-dimensional (1D) CC/TiO_2_ nanotube arrays. Subsequently, ultrathin FeOOH nanosheets were grown on the surface of the CC/TiO_2_ nanotube arrays and then spontaneously transformed into hollow nanospheres by the forced hydrolysis of FeCl_3_, without the use of any structure-directing agent. FeOOH hollow nanospheres grown on the CC/TiO_2_ nanotube arrays were further transformed into α-Fe_2_O_3_ hollow nanospheres through thermal dehydroxylation and lattice shrinkage at a high temperature, leading to the formation of the charantia-like CC/TiO_2_/Fe_2_O_3_ nanotube arrays.

**Figure 1:**
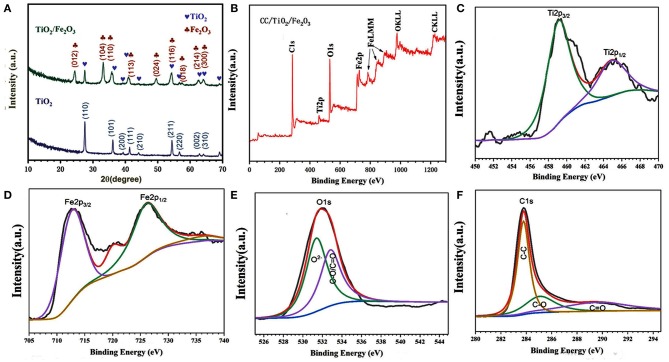
**(A)** XRD pattern of CC/TiO_2_ and CC/TiO_2_/Fe_2_O_3_ hollow nano-arrays. XPS spectra of CC/TiO_2_/Fe_2_O_3_ specimens. **(B)** Survey spectra. **(C)** Ti2p core-level spectra, **(D)** Fe2p core-level spectra. **(E)** O1s core-level spectra, and **(F)** C1s core-level spectra.

XRD measurements were performed to confirm the crystalline structure of the obtained products. Figure 1A shows the corresponding XRD patterns of TiO_2_ and TiO_2_/Fe_2_O_3_ products scratched from CC substrates. All the diffraction peaks of the TiO_2_ products matched well with those of typical tetragonal rutile TiO_2_ (JCPDS No. 21-1272). The lattice parameters of the obtained tetragonal rutile TiO_2_ nanotube are a = b = 4.516 Å and c = 5.205 Å. These are consistent with those reported by Luo et al. (2012). Three additional peaks were observed in the XRD pattern of the TiO_2_/Fe_2_O_3_ composite. These peaks located at 33.1, 35.6, and 54.1° correspond to the (104), (110), and (116) planes of α-Fe_2_O_3_ (JCPDS No. 33-0664), respectively, indicating the successful preparation of an α-Fe_2_O_3_ shell on the TiO_2_ nanotube core (Larcher et al., 2003; Qi et al., 2018).

XPS was performed to identify the chemical states and surface composition of the final composite. The spectra are shown in Figures 1B–F. Only the peaks of Ti, O, Fe, and C are found in the survey spectrum of the CC/TiO_2_/Fe_2_O_3_ composite. The XPS spectrum of the C 1s core-level could be divided into three peaks (Figure 1C). The peak at 283.7 eV indicates the non-oxygenated carbon. The other two peaks at 284.9 and 289.1 eV correspond to the carbon ramifications generated in the hydrothermal reaction and annealing process. The deconvoluted peaks of the O 1s spectrum could also be decomposed into two components centered at 531.3 and 533.8 eV, respectively. The low binding energy component is attributed to the ${\text{O}}_{2}^{-}$ attached to Ti and Fe, the other component corresponds to the oxygen in C–O and O–C=O bonds. The Ti 2p XPS spectrum exhibits bimodal characteristics. The peaks located at 460 and 466 eV correspond to Ti 2p_3/2_ and Ti 2p_1/2_, respectively (Wang et al., 2016). The binding energy difference of 5.8 eV between the Ti 2p_1/2_ and Ti 2p_3/2_ core levels indicated that the oxidation state of Ti is mainly Ti (4+). Similarly, the typical bimodal characteristics of the Fe 2p XPS spectrum also confirmed the existence of surface Fe as a trivalent oxide (Luo et al., 2012; Yang et al., 2017). Consistent with the results of XRD, the XPS analysis revealed that the obtained composites are composed of α-Fe_2_O_3_ and TiO_2_.

**Figure 2:**
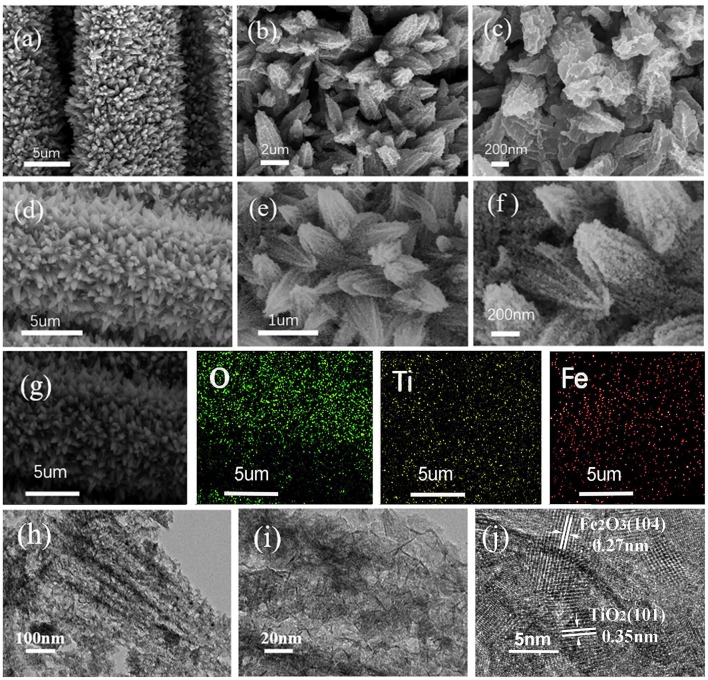
SEM images of the prepared specimens at different magnifications. **(a–c)** CC/TiO_2_ specimens. **(d–f)** CC/TiO_2_/Fe_2_O_3_ specimens. **(g)** EDX elemental mapping of the CC/TiO_2_/Fe_2_O_3_ specimens. **(h–j)** TEM and HRTEM images of CC/TiO_2_/Fe_2_O_3_ specimens.

The morphology and microstructure of the as-obtained composite were characterized by SEM and TEM. Figures 2a–c show the SEM images of pure CC/TiO_2_ nanotube arrays at different magnifications. It is obvious that the 1D TiO_2_ nanotube arrays grew uniformly and vertically on the CC substrate in a carambola-like configuration. The high-magnification SEM images clearly show that the 1D carambola-like TiO_2_ nanotube arrays have a mean bottom diameter of 500 nm. Their walls were assembled from several small nanosheets, leading to the roughening of the surface of the TiO_2_ nanotube arrays. A rough surface facilitates the heterogeneous nucleation of Fe_2_O_3_ nanoparticles. Figures 2d–f present the SEM images of CC/TiO_2_/Fe_2_O_3_ nanocomposite arrays at different magnifications. As seen, a large number of nanospheres adhered to the surface of the carambola-like TiO_2_ nanotube arrays, forming a 3D hierarchical charantia-like structure. This association rendered the final nanotube arrays more compact and spatially dense, thus guaranteeing a high mass loading and high specific capacity. The mass loading of CC/TiO_2_/Fe_2_O_3_ nanocomposite arrays was ~11.6 mg cm–^2^, higher than that of the pure TiO_2_/CC nano-arrays (~5.1 mg cm^−2^). Owing to these features, the final nanotube arrays can remain ordered without undergoing any apparent structural collapse or breakage. Compared with the bare TiO_2_ nanotube arrays, the bottom diameters of the 3D hierarchical CC/TiO_2_/Fe_2_O_3_ nano-composite arrays range up to 700 nm, with negligible variation in the length. The surface of the charantia-like CC/TiO_2_/Fe_2_O_3_ nanotube arrays is rougher and looser than that of the 1D bare CC/carambola-like TiO_2_ nanotube arrays. These characteristics improved the effective contact area with the electrolyte. Furthermore, the robust bottom of the charantia-like nano-composite arrays increased their mechanical strength, thus reducing the fracture risk triggered by volume variations during lithiation/delithiation. This unique structure may lead to a large specific capacity and high cycling stability of the final product (Joshi et al., 2016; Zhang et al., 2019). Figure 2g displays the EDX mapping analysis of the charantia-like CC/TiO_2_/Fe_2_O_3_ nanotube arrays. Fe, Ti, and O are evenly distributed on the surface of carbon fibers, further implying that the nanospheres coated on the surface of 1D TiO_2_ nanotube arrays are iron oxides.

To investigate the microstructure of the charantia-like nanotube arrays, further TEM observations were made. Figures 2h–j show the TEM images of the nanotube arrays scraped off the CC substrate. Figure 2h shows that both the TiO_2_ cores and Fe_2_O_3_ nanospheres have hollow interiors. The hollow Fe_2_O_3_ nanospheres are loosely assembled from numerous small nanosheets, indicating that they might have a mesoporous structure. Further, the HRTEM image shows that the lattice spacings of the 3D hierarchical charantia-like CC/TiO_2_/Fe_2_O_3_ nanotube arrays are 0.35 and 0.27 nm, corresponding to the (101) plane of tetragonal rutile TiO_2_ and the (110) plane of α-Fe_2_O_3_, respectively (Luo et al., 2012; Qi et al., 2018). This result further confirms that the nanotube arrays were composed of tetragonal rutile TiO_2_ and α-Fe_2_O_3_.

**Figure 3:**
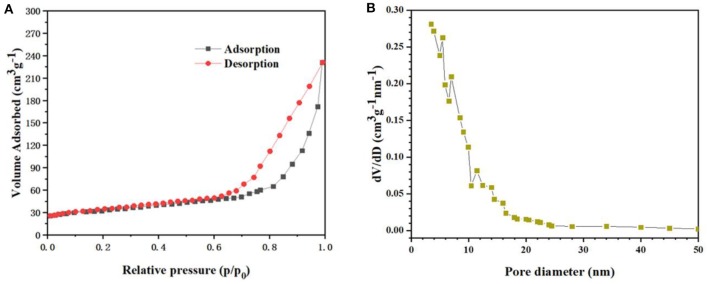
**(A)** The N_2_ adsorption/desorption isotherms and **(B)** pore size distribution of CC/TiO_2_/Fe_2_O_3_ hollow nano-arrays.

BET gas-sorption measurements were performed to further investigate the porous nature and the specific surface area of the nanotube arrays. Figure 3A presents the nitrogen adsorption–desorption isotherm of the nanotube arrays. The typical IV isotherm with a distinct hysteresis loop in the range of 0.7–1.0 P/Po indicates that the charantia-like TiO_2_/Fe_2_O_3_ nanotube arrays have a mesoporous structure (Luo et al., 2013; Zhang et al., 2019). The BET specific surface area of the nanotube arrays was calculated to be 149.3 m^2^ g^−1^. The large specific surface area is mainly due to the extremely uneven charantia-like surface resulting from the well-wrapped Fe_2_O_3_ hollow nanospheres. The pore size distribution (Figure 3B) demonstrates that the main pore size of the nanotube arrays is approximately 13.4 nm. According to the literature (Luo et al., 2012; Wang et al., 2016; Zhang et al., 2019), such a mesoporous structure can offer enough interface for lithium-ion insertion and extraction.

### Electrochemical Properties

**Figure 4:**
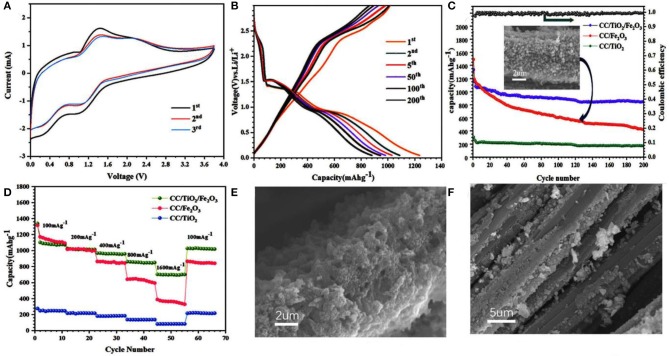
**(A)** CV curves of CC/TiO_2_/Fe_2_O_3_ specimens between 5 mV and 3.8 V at a scan rate of 0.5 mVs^−1^. **(B)** Charge-discharge voltage profiles of CC/TiO_2_/Fe_2_O_3_ specimens at a constant current rate of 200 mAg^−1^. **(C)** Cycling performances of CC/TiO_2_/Fe_2_O_3_, CC/TiO_2_, and CC/Fe_2_O_3_ specimens at a constant current rate of 200 mA g^−1^. **(D)** Rate performances of CC/TiO_2_/Fe_2_O_3_, CC/TiO_2_, and CC/Fe_2_O_3_ specimens. SEM image of **(E)** CC/TiO_2_/Fe_2_O_3_ and **(F)** CC/Fe_2_O_3_ specimens after 200 cycles.

The electrochemical properties of the 3D hierarchical charantia-like CC/TiO_2_/Fe_2_O_3_ nanotube arrays were evaluated to further confirm the performance of the prepared nanotube arrays. Figure 4A shows the first three CV cycles of a single redox couple in the voltage window of 0–3.8 V at a scanning rate of 0.5 mV/s. The first CV curve shows two broad cathodic peaks at 1.05 and 0.42 V, which mainly correspond to the generation of Li_2_O as well as the formation of a solid-electrolyte interface (SEI) (Luo et al., 2012; Yang et al., 2017). In the anodic process, the peak at 1.4 V indicates the oxidation of zero-valent iron to ferric iron, while the weak peak at 2.3 V is ascribed to the delithiation of TiO_2_. Except for the slight variation in the two cathodic peaks, the subsequent curves exhibit good reproducibility. The slight shift in the two cathodic peaks to higher potential reveal the occurrence of some irreversible processes during the first cycle. This result is consistent with the previous findings reported in literature (Luo et al., 2012; Qi et al., 2018; Zhang et al., 2019).

Figure 4B shows different cycle charge–discharge voltage profiles of the 3D hierarchical charantia-like CC/TiO_2_/Fe_2_O_3_ nanotube arrays at a constant current density of 200 mA g^−1^. The plateau voltages in the first discharge curve are in good agreement with the oxidation peaks in the CV curves. The initial discharge and charge capacities are ~1,280 and 1,089 mAh g^−1^, respectively, corresponding to a low irreversible capacity loss (~15%). Figure 4C shows the cycling performances of the CC/TiO_2_/Fe_2_O_3_ nanotube arrays as along with those of CC/TiO_2_ nanotube arrays and CC/Fe_2_O_3_ composites. The CC/Fe_2_O_3_ composites were synthesized from clean CC using the same Fe ion hydrothermal system mentioned in the experimental section. Although the bare CC/TiO_2_ nanotube arrays presented a perfect cycle performance, they had a low capacity. On the contrary, despite having a high capacity in the initial stage, the CC/Fe_2_O_3_ composite showed a rapid decay of the capacity with cycling. The apparent decline in the capacity is mainly due to the drastic volume changes of Fe_2_O_3_ itself during lithiation, which led to the removal of the active material from the CC substrate. By contrast, the combination of low volume expansion of the TiO_2_ hollow cores and the strong adhesion between the two types of metallic oxides rendered the structure of the 3D nanotube arrays stable during lithium-ion insertion/extraction processes. This feature further led to high cyclic capacity retention of the nanotube arrays. Even after 200 cycles, the discharge capacity of the CC/TiO_2_/Fe_2_O_3_ nanotube arrays was maintained at approximately 896 mAh g^−1^, which is much higher than that of the bare CC/TiO_2_ nanotube arrays and CC/Fe_2_O_3_ composites. At the 200th cycle, the Coulombic efficiency of the nanotube array electrodes was approximately 98.7%. Figure 4D displays the rate performance of the tested electrodes in the rate range of 100–1,600 mA g^−1^. The CC/TiO_2_/Fe_2_O_3_ nanotube array electrode maintained a discharge capacity of approximately 742 mAh g^−1^ even when the discharge rate was increased to 1,600 mA g^−1^. Meanwhile, the discharge capacity of the CC/Fe_2_O_3_ composite electrode was only approximately 397.5 mA g^−1^ at 1,600 mA g^−1^. Compared the previous literature (Luo et al., 2012, 2013; Xia et al., 2014), these charantia-like nanotube arrays exhibit superior capacity and cyclability due to their unique double hollow structure (Table 1).

**Table 1:** List of research reports on TiO_2_/Fe_2_O_3_ as lithium-ion anodes.

**Materials**	**Capacity/constant current density**	**Cycles**	**References**
CC/TiO_2_/Fe_2_O_3_ nanotube arrays	896 mAh g^−1^, 200 mA g^−1^	200 cycles	This work
TiO_2_@a-Fe_2_O_3_ on carbon textiles	480 mAh g^−1^, 120 mA g^−1^	150 cycles	Luo et al., 2012
TiO_2_@α-Fe_2_O_3_ carbon coated	516 mAh g^−1^, 200 mA g^−1^	200 cycles	Luo et al., 2013
TiO_2_-B@α-Fe_2_O_3_	785 mAh g^−1^, 100 mA g^−1^	100 cycles	Xia et al., 2014
TiO_2_@α-Fe_2_O_3_	600 mAh g^−1^, 200 mA g^−1^	200 cycles	Luo et al., 2013

Figures 4E,F illustrate the SEM images of the CC/TiO_2_/Fe_2_O_3_ nanotube array and the CC/Fe_2_O_3_ composite electrodes after 200 cycles. The former maintained an integral framework and was tightly adsorbed to the surface of the CC substrate after 200 cycles. Under the same condition, the volume of Fe_2_O_3_ itself changed unprecedentedly upon cycling, resulting in a large alternate stress. As a result, the CC/Fe_2_O_3_ composite severely fragmented and extensively separated from the CC substrate. Consistent with the charge–discharge test results, the electrical contact loss significantly reduced the cycling life of the CC/Fe_2_O_3_ composite electrode.

**Figure 5:**
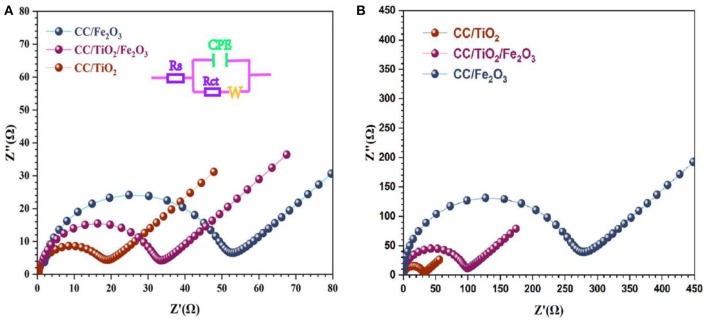
Impedance analysis of CC/TiO_2_/Fe_2_O_3_, s CC/TiO_2_, and CC/Fe_2_O_3_ specimens for **(A)** fresh cell and **(B)** 50th cycled cell.

To gain further insight into the electrochemical performance of the test specimens, EIS measurements were carried out at room temperature. Figures 5A,B show the Nyquist plots of the test specimens at their open-circuit voltage. All the test specimens exhibited semi-circular Nyquist plots in the high-frequency region and a straight line in the low-frequency region. The diameter of the semicircle represents the charge-transfer resistance (*R*_CT_), while the slope of the line corresponds to the mass transfer of lithium ions (*W*) (Shen et al., 2019; Zhang et al., 2019). By adding the Ohmic resistance (*R*_s_) and double-layer capacitance (CPE), the electrochemical system can be fitted to the Randles circuit shown in Figure 5A. As shown in Figure 5, the diameters of the semicircle of the 3D hierarchical charantia-like CC/TiO_2_/Fe_2_O_3_ nanotube arrays and bare CC/TiO_2_ nano-arrays are smaller than that of the CC/Fe_2_O_3_ composite, indicating that the conductivity of the hollow CC/TiO_2_/Fe_2_O_3_ nano-arrays can be effectively improved by their TiO_2_ nanotube cores. After 200 cycles, the charge-transfer resistance of the CC/TiO_2_/Fe_2_O_3_ nanotube arrays and bare CC/TiO_2_ nanotube arrays increased marginally, while that of the CC/Fe_2_O_3_ composites increased significantly.

## Conclusions

3D hierarchical charantia-like TiO_2_/Fe_2_O_3_ nanotube arrays were fabricated on CC by a simple, two-step, seed-free hydrothermal method. The well-wrapped mesoporous Fe_2_O_3_ nanospheres on TiO_2_ nanotubes endowed the final nanotube arrays with a larger specific surface area. The hollow framework and its direct contact with the CC current collector led to the good conductivity of the electrode. The low volume expansion of the hollow TiO_2_ cores combined with the strong adhesion between the two types of metal oxides stabilized the structure of the final nanotube arrays during cycling. The electrochemical results revealed that the charantia-like TiO_2_/Fe_2_O_3_ nanotube arrays have high reversible capacity, improved cycling stability, and excellent rate capability. After 200 cycles at a current density of 200 mA g^−1^, the capacity of the nanotube arrays remained at 875 mAh g^−1^. A rate capability of ~742 mAh g^−1^ was achieved even when the discharge rate was increased to 1,600 mA g^−1^.

## Data Availability Statement

The datasets generated for this study are available on request to the corresponding author.

## Author Contributions

PX, HZ, AS, and YZha conducted the synthesis. PX, YZha, and YW carried out the characterization and the electrochemical measurements. PX and ZZ co-wrote the manuscript. All authors discussed the data and commented on the manuscript.

### Conflict of Interest

The authors declare that the research was conducted in the absence of any commercial or financial relationships that could be construed as a potential conflict of interest.
